# Salt-Induced Changes in Cardiac Phosphoproteome in a Rat Model of Chronic Renal Failure

**DOI:** 10.1371/journal.pone.0100331

**Published:** 2014-06-19

**Authors:** Zhengxiu Su, Hongguo Zhu, Menghuan Zhang, Liangliang Wang, Hanchang He, Shaoling Jiang, Fan Fan Hou, Aiqing Li

**Affiliations:** Division of Nephrology, Nanfang Hospital, Southern Medical University, State Key Laboratory of Organ Failure Research, National Clinical Research Center of Kidney Disease, Guangzhou, Guangdong, China; Rouen University Hospital, France

## Abstract

Heart damage is widely present in patients with chronic kidney disease. Salt diet is the most important environmental factor affecting development of chronic renal failure and cardiovascular diseases. The proteins involved in chronic kidney disease -induced heart damage, especially their posttranslational modifications, remain largely unknown to date. Sprague-Dawley rats underwent 5/6 nephrectomy (chronic renal failure model) or sham operation were treated for 2 weeks with a normal-(0.4% NaCl), or high-salt (4% NaCl) diet. We employed TiO_2_ enrichment, iTRAQ labeling and liquid-chromatography tandem mass spectrometry strategy for phosphoproteomic profiling of left ventricular free walls in these animals. A total of 1724 unique phosphopeptides representing 2551 non-redundant phosphorylation sites corresponding to 763 phosphoproteins were identified. During normal salt feeding, 89 (54%) phosphopeptides upregulated and 76 (46%) phosphopeptides downregulated in chronic renal failure rats relative to sham rats. In chronic renal failure rats, high salt intake induced upregulation of 84 (49%) phosphopeptides and downregulation of 88 (51%) phosphopeptides. Database searches revealed that most of the identified phospholproteins were important signaling molecules such as protein kinases, receptors and phosphatases. These phospholproteins were involved in energy metabolism, cell communication, cell differentiation, cell death and other biological processes. The Search Tool for the Retrieval of Interacting Genes analysis revealed functional links among 15 significantly regulated phosphoproteins in chronic renal failure rats compared to sham group, and 23 altered phosphoproteins induced by high salt intake. The altered phosphorylation levels of two proteins involved in heart damage, lamin A and phospholamban were validated. Expression of the downstream genes of these two proteins, desmin and SERCA2a, were also analyzed.

## Introduction

Chronic kidney disease (CKD) is a global public health problem affecting over 10.8% or 13% of western [1] or Chinese population, respectively [2]. A large number of observational studies have demonstrated excess cardiovascular risks associated with CKD [3]–[5]. Rate of cardiovascular morbidity and mortality significantly increased in adults with CKD as compared with general population [3], [6]. Conventional cardiovascular risk factors such as hypertension and diabetes are highly prevalent in patients with CKD and end-stage renal disease. Cardiovascular diseases occur in progressive stages of chronic renal failure[7] , in which besides the conventional cardiovascular risk factors, many factors more specific to CKD, such as proteinuria, anaemia, left ventricular hypertrophy, arterial calcification, abnormal calcium/phosphate/vitamin D homeostasis and inflammation contribute to cardiovascular risk [8]. Heart damage is widely present in patients with CKD, but the mechanisms underlying CKD-induced heart damage remains unclear.

Numerous epidemiologic, clinical, and experimental studies demonstrate dietary salt intake has been related to blood pressure, and salt restriction has been documented to lower blood pressure [9], [10]. Patients with CKD often are salt sensitive and their blood pressure increased with increasing salt intake [11]. Hypertension is common in non-dialysis CKD patients and known as a major risk factor for CVD as well as progression of renal disease [12], [13]. Cardiovascular events occurred more frequently in patients with salt-sensitive hypertension. Salt sensitivity has been demonstrated an independent cardiovascular risk factor in Japanese patients with essential hypertension [14]. In contraste, sodium reduction, may reduce long term risk of cardiovascular events [15]. In addition, left ventricular hypertrophy and pulse pressure were influenced by salt intake independent of blood pressure in humans [16]–[18]. Together, salt diet is the most important environmental factor affecting the development of chronic renal failure and cardiovascular diseases.

Protein phosphorylation is a ubiquitous post-translational modification involved in several key intracellular processes including metabolism, secretion, homeostasis, transcriptional and translational regulation, and cellular signaling [19]. There is overwhelming evidence that protein phosphorylation plays a critical role in cardiac remodeling process. First, a lot of serine–threonine kinases and kinase signaling pathways, such as PI3K, Akt, GSK-3, TGF-β, CaMKII, PkA, MAPKs, PkC, etc., are involved in regulation of cardiovascular diseases [20]–[22]; Second, secretion and generation of vasoconstrictor peptides, such as angiotensin II, endothelin-1, norepinephrine, and Rho and Ras proteins, are increased through the activation of protein kinases [22], [23] and play critical roles in hypertrophic response to nephrogenic hypertension; Third, protein phosphatase such as protein phosphatase 1 and calcineurin, and a number of phosphoproteins such as phospholamban and epidermal growth factor receptor, are also involved in the remodeling process [22]–[24]. Mass spectrometry (MS)-based proteomics in combination with phosphoprotein enrichment technique is to-date probably the most powerful tool to analyze large-scale protein phosphorylation events in a variety of biological samples without a prior knowledge of function or distribution [25]. However, there are so far no studies on heart phosphoproteomic change associated with CKD.

In this study, we performed large-scale phosphoproteomic analysis of left ventricular free walls in a salt-load rat model of chronic renal failure using tandem MS [liquid chromatography (LC)−MS/MS] mechods used previously [26]–[28] along with TiO_2_ enrichment. We identified a total of 1724 unique phosphopeptides, including 165 and 132 phosphopeptides differentially regulated in chronic renal failure and by high salt intake, respectively. This study provides a database resource for future studies of heart diseases. We hope that new scientific research ideas and therapeutic strategies deriving from phosphoproteins or phosphorylation sites reported in this study could be employed to antagonize heart diseases either with or without renal disease.

## Materials and Methods

### Ethics Statement

The care and use of the rats were approved by the Animal Experiment Ethics Committee of Southern Medical University.

### Animals

Male Sprague-Dawley rats (initial weight 150 to 180 g; Southern Medical University Animal Experiment Center) were maintained under standardized conditions and fed a standard rodent diet that contained 16% protein. The rats were divided into three groups. Briefly, the rats were subjected either to five-sixths nephrectomy (5/6 Nx; *n* = 12; by performing a right nephrectomy with surgical resection of two thirds of the left kidney) or to sham operation (controls; *n* = 6). One week after the operation, the 5/6 Nx rats were randomized by the percent remnant kidney weight removed ([right kidney weight − weight of two poles of left kidney]/right kidney weight×100) and were divided into two subgroups (n = 6 in each group). At the end of 4, 8, and 10 wk after operation, the rats (n = 6 in each group at each time point) were anesthetized with sodium pentobarbital and Orbital venous blood was collected from the 5/6 Nx and sham rats for hemodynamic detection.

**Figure 1:**
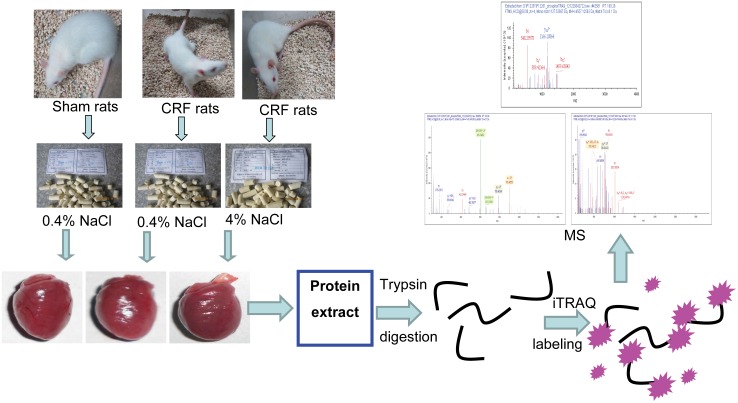
Flow chart of phosphoproteomic analysis of left ventricle free walls in sham and chronic renal failure rats. Male Sprague-Dawley rats were subjected either to five-sixths nephrectomy or to sham operation. Ten weeks after surgery, 5/6 nephrectomy induced chronic renal failure (CRF) rats were fed normal salt (0.04% NaCl) or high salt (4% NaCl) diet for 2 weeks. Sham group was maintained with normal salt diet. The whole heart was harvested and the free wall of left ventricle was dissected for protein extraction. The proteins were then digested with trypsin, labeled using the 8-plex isobaric tags for relative and absolute quantification (iTRAQ) reagent and multiplexed. The phosphorylated peptides enriched with TiO_2_ beads were subjected for nano-liquid chromatography tandem mass spectrometry (MS) analysis using a Q Exactive MS equipped with easy nano-liquid chromatography.

The experimental procedures are illustrated in Figure 1.

### Salt Diet Treatment and Tissue Preparation

At the end of week 10 after operation, 5/6 nephrectomy rats and sham rats were randomly divided into 3 groups and treated as follows: (1) sham-operated rats with normal-salt diet (0.4% sodium chloride, wt/wt) (NS, n = 6); (2) 5/6 nephrectomy rats with normal-salt diet (0.4% sodium chloride, wt/wt) (NC, n = 6); (3) 5/6 nephrectomy rats with high-salt diet (4% sodium chloride, wt/wt) (HC, n = 6). The rats received commercially available rat chow containing different concentrations of salt (TROPHIC, Nantong, China) for 2 weeks. After salt diet administration, the whole heart was harvested after perfusion with 200 ml of ice-cold normal saline and washed with normal saline. The harvested heart was then weighed, and the upper third part of the left ventricle was dissected for histological analysis. After removing the atria and right ventricle, the free wall of left ventricle was quickly placed in liquid nitrogen until protein or RNA extraction. The 24-h urine samples were collected in metabolic cages at end of the study period.

### Renal Function and BP Measurement

Serum and urine creatinine levels were determined using commercial kits (sarcosine oxidase-peroxidase-antiperoxidase; Zixing, Shanghai, China). The creatinine clearance (Ccr) was calculated as described previously and factored for body weight [29]. The 24-hr urinary protein excretion was measured using the Coomassie Blue method [30].

Blood pressure was measured using tail cuff with a sphygmomanometer (BP-98A, softron, Japan) before and after salt diet treatment. Systolic blood pressure was measured 5–6 times and the values were averaged.

### Protein Extraction

Approximately 2 g frozen, treated heart tissues from an equal amount of four biological replicates in the same subgroup were ground into a powder in liquid nitrogen and homogenized in extraction buffer [4% SDS, 1 mM DTT, 150 mM Tris-HCl, pH 8]. After 3 min incubation in boiling water, the homogenate was sonicated on ice. The crude extract was then incubated in boiling water again and clarified by centrifugation at 16,000 g at 25°C for 10 min. The protein content was determined by the Bicinchoninic acid protein assay kit (Beyotime, China).

### Protein Digestion and iTRAQ Labeling

Protein digestion was performed according to the FASP procedure described by Wisniewski et al. [31] and the resulting peptide mixture was labeled using the 8-plex iTRAQ (isobaric tags for relative and absolute quantification) reagent according to the manufacturer’s instructions (Applied Biosystems). Briefly, 200 µg of proteins for each sample were incorporated into 30 µl standard buffer (4% SDS, 100 mM DTT, 150 mM Tris-HCl pH 8.0). The detergent, DTT and other low-molecular-weight components were removed using uric acid (UA) buffer (8 M Urea, 150 mM Tris-HCl pH 8.0) by repeated ultrafiltration (Microcon units, 30 kD). Then 100 µl 0.05 M iodoacetamide in UA buffer was added to block reduced cysteine residues and the samples were incubated for 20 min in darkness. The filters were washed with 100 µl UA buffer three times and then 100 µl DS buffer (50 mM triethylammoniumbicarbonate at pH 8.5) twice. Finally, the protein suspensions were digested with 2 µg trypsin (Promega) in 40 µl DS buffer overnight at 37°C, and the resulting peptides were collected as a filtrate. The peptide content was estimated by UV light spectral density at 280 nm using an extinctions coefficient of 1.1 of 0.1% (g/l) solution that was calculated on the basis of the frequency of tryptophan and tyrosine in vertebrate proteins. For labeling, each iTRAQ reagent was dissolved in 70 µl of ethanol and added to the respective peptide mixture. The samples marked NS, NC and HC were labeled with iTRAQ tags 113, 114 and 115, respectively, multiplexed and vacuum dried.

### Enrichment of Phosphorylated Peptiedes by the TiO_2_ Beads

The final peptide mixture, which was concentrated by a vacuum concentrator, was resuspended in 500 µL loading buffer (2% glutamic acid/65% ACN/2% TFA). Then, TiO_2_ beads were added and then agitated for 40 min. The centrifugation was carried out for 1 min at 5000 g, resulting in the first beads. The supernatant from the first centrifugation were mixed with another TiO_2_ beads, resulting in the second beads which collected as before. Both beads were combined and washed with 50 uL of washing buffer I (30% ACN/3% TFA) three times and then 50 µL of washing buffer II (80% ACN/0.3% TFA) three times to remove the remaining non-adsorbed material. Finally, the phosphopeptides were eluted with 50 uL of elution buffer (40% ACN/15% NH_4_OH), followed by lyophilization and MS analysis.

### Mass Spectrometry

Five microliters of the phosphopeptides solution mixed with 15 ul 0.1% (v/v) trifluoroacetic acid and then 10 ul of the solution mixture was injected for nanoLC-MS/MS analysis using an Q Exactive MS (Thermo Finnigan) equipped with Easy nLC (Proxeon Biosystems, now Thermo Fisher Scientific). The peptide mixture was loaded onto a C18-reversed phase column (15 cm long, 75 µm inner diameter, RP-C18 3 µm, packed in-house) in buffer A (0.1% formic acid) and separated with a linear gradient of buffer B (80% acetonitrile and 0.1% formic acid) at a flow rate of 250 nL/min controlled by IntelliFlow technology over 240 min. The peptides were eluted with a gradient of 0%–60% buffer B from 0 min to 200 min, 60% to 100% buffer B from 200 min to 216 min, 100% buffer B from 216 min to 240 min.

For MS analysis, peptides were analyzed in positive ion mode. MS spectra were acquired using a data-dependent top 10 method dynamically choosing the most abundant precursor ions from the survey scan (300–1800 m/z) for higher energy collisional (C-trap) dissociation (HCD) fragmentation. Determination of the target value is based on predictive Automatic Gain Control (pAGC). Dynamic exclusion duration was 40.0 s. Survey scans were acquired at a resolution of 70,000 at m/z 200 and resolution for HCD spectra was set to 17,500 at m/z 200. Normalized collision energy was 27 eV and the under fill ratio, which specifies the minimum percentage of the target value likely to be reached at maximum fill time, was defined as 0.1%. The instrument was run with peptide recognition mode enabled.

### Data Analysis

MS/MS spectra were searched using Mascot 2.2 engine against the Uniprot database and the reversed database. For protein identification, the following options were used. Peptide mass tolerance = 20 ppm, MS/MS tolerance = 0.1 Da, Enzyme = Trypsin, Missed cleavage = 2, Fixed modification: Carbamidomethyl (C), Variable modification: Oxidation (M), Phosphorylation (S/T/Y), FDR≤0.01.

The phosphorylation peptides were analyzed using Proteome Discoverer 1.3 (Thermo Electron, San Jose, CA). pRS score above 50 indicate a good PSM (Peptide Spectrum Matches) and pRS probabilities above 75 percent indicate that a site is truly phosphorylated.

### Western Blot Analysis

Western blot analyses were carried out as described previously [26]. Briefly, left ventricular free walls were lysed with the radioimmunoprecipitation assay buffer, and the lysates were separated by SDS-PAGE. The separated proteins were then transferred to Polyvinylidene fluoride membranes. The membrane blots were first probed with a primary antibody overnight at 4°C and then secondary antibody coupled to horseradish peroxidase. The proteins were visualized with the enhanced chemiluminescent system (Pierce, Rockford, IL) and the bands densitometry was analyzed. Phosphoprotein levels were normalized to total protein levels. The primary antibodies used included anti-Phospho-phospholamban (Ser16, Cell Signaling, Beverly, MA, USA), anti-phospholamban (Abcam, Cambridge, MA, USA), anti-Phospho-lamin A (Ser22, Santa Cruz Biotechnology, Santa Cruz, CA, USA) and anti-lamin A (Abcam).

### Real-time Reverse Transcriptase-polymerase Chain Reaction

Total RNA was extracted from left ventricular free walls in the animals using Trizol reagent (Invitrogen). Aliquots of each RNA extraction were reverse-transcribed simultaneously into cDNA using M-MLV reverse transcriptase according to the manufacturer’s protocol (Invitrogen). Each quantitative real-time PCR was performed in a total volume of 25 µL in duplicate by using the Premix Ex Taq kit (TaKaRa, Kyoto, Japan) and the Fast Real-Time PCR system 7500 (Applied Biosystems, CA). The thermal cycling conditions comprised a 30-second step at 95°C, followed by 40 cycles with denaturation at 95°C for 5 seconds, annealing at 60°C (desmin, SERCA2) or 56°C (GAPDH)) for 30 seconds, and extension at 72°C for 60 seconds. The following sets of primers, which were designed using Primer Quest software, were used: desmin forward: 5′–GGG CGA GGA GAG CCG GAT CA–3′, reverse: 5′–TCC CCG TCC CGG GTC TCA ATG–3′; SERCA2 forward: 5′–AAG CAG TTC ATC CGC TAC CT–3′, reverse: 5′–AGA CCA TCC GTC ACC AGA TT–3′; GADPH forward: 5′–GGG TGT GAA CCA CGA GAA AT–3′, resverse: 5′–ACT GTG GTC ATG AGC CCT TC–3′. For normalization of differences in RNA amounts, the GAPDH RNA was coamplified. Relative quantification of each gene was calculated after normalization to GAPDH RNA by using the comparative Ct method. The results were shown as relative expression ratio with respect to NC group for all samples.

## Result

### Physiological Parameters

Ten weeks after 5/6 nephrectomy or sham operation, 5/6 Nx rats displayed substantially elevated systolic blood pressure (SBP), serum creatinine, blood urea nitrogen and 24-hour urinary protein excretion relative to sham rats, that demonstrated chronic renal failure (CRF) rats were successfully prepared. The rats were fed high or normal salt diets for 2 weeks (see Materials and Methods). As shown in Table 1, high salt intake induced a significant increase in SBP, urinary sodium excretion and urinary protein excretion in CRF rats relative to normal salt intake, suggesting that high salt intake aggravated kidney damage. Both the average heart weight and heart weight/body weight ratio of CRF rats with high salt diet was significantly greater than that with normal salt diet. These data demonstrated that high salt intake aggravated cardiac hypertrophy in CRF rats.

**Table 1:** Physiological and metabolic parameters in Sham and CRF rats at week 12 after surgery.^A^

	Sham	CRF	
	Normal salt	Normal salt	High salt
HW (mg)	1.6±0.1	1.9±0.1^B^	2.3±0.1^C^
HW/BW (*1000)	2.9±0.0	3.8±0.1^B^	4.2±0.2^C^
SBP (mmHg)	126.3±4.1	137.1±3.3^B^	153.1±3.5^C^
Serum Na^+^(mmol/l)	139.6±0.8	141.3±1.3	144.3±0.37^C^
Urine Na^+^ µmol/24 h)	871.0±67.9	747.0±69.3	11212.2±1012.2^C^
UPE (mg/24 h)	10.60±0.7	16.38±1.2^B^	40.29±3.1^C^

A Data from 3 independent experiments are expressed as mean ± SD (n = 6 in each group);

B *P*<0.05 versus rats fed with normal salt in the sham group;

C *P<*0.05 versus CRF group fed with normal salt diet.

HW, heart weight, recorded for perfused hearts after removal of the atria and major blood vessels; HW/BW, heart weight/body weight; SBP, systolic blood pressure; UPE, urinary protein excretion.

### Identification of Phosphorylated Proteins and Sites

**Figure 2:**
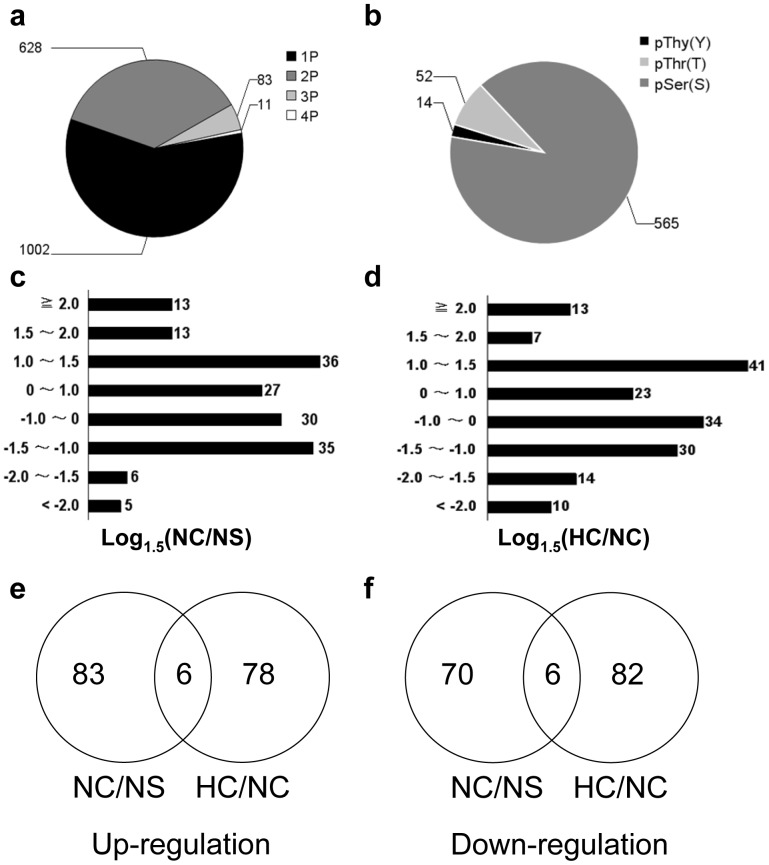
Characterization of phosphopeptides, phosphosites and phosphoproteins. (a) Distribution of singly, doubly, triply and quadruply phosphorylated peptides; (b) Distribution of the Ser, Thr, Tyr phosphosites in the heart phosphoproteome. Phosphopeptides were categorized into class I phosphosites by calculating the probabilities for phosphorylation at each site based on posttranslational modification scores. Here, only class I phosphosites (high probability) were used to analyze the distribution. (c, d) Phosphopeptide Log 1.5 (NC/NS) and Log 1.5 (HC/NC) ratio after different salt intake. (e, f) Venn diagram of differentially phosphorylated peptides in NC/NS and HC/NC comparison groups. HC represents CRF rats with high salt intake, NC represents CRF rats with normal salt intake. NS represents sham rats with normal salt intake. 1P, 2P, 3P and 4P represent singly, doubly, triply and quadruply phosphorylated peptides, respectively. NS, sham-operated rats fed with normal-salt diet; NC, 5/6 nephrectomized rats fed with normal-salt diet; HC, 5/6 nephrectomized rats fed with high-salt diet.

In this study, phosphopeptides were identified after manual confirmation of MS/MS spectra by combining phosphopeptide enrichment using titanium dioxide with LC−MS/MS quantitative proteomics using iTRAQ. These identified phosphopeptides (Table S1) were clustered into 1724 unique peptides representing 2551 non-redundant phosphorylation sites on 763 different proteins. To precisely assign phosphorylation sites within a peptide, we used posttranslational modification score to calculate probabilities of phosphorylation at each site as previously described [32]. We could localize 1002 phosphosites with high confidence as class I phosphorylation site, i.e., singly-phosphorylated. Around 58.1% of the phosphopeptides identified were found to be singly phosphorylated including 14 phosphotyrosine sites, 52 phosphothreonine sites, and 565 phosphoserine sites. The other peptides were doubly (36.4%), triply (4.8%), or more highly (0.6%) phosphorylated (Figure 2 a and b). It is worth pointing out that most of proteins identified from the phosphopeptides are important signaling molecules such as protein kinases, receptors, phosphatases, and transcription regulators including transcription factors and repressors. They are involved in cell energy metabolism, signal transduction, apoptosis and other biological processes.

Among these differentially phosphorylated peptides (Table S2), we found that ∼6.3% in NC versus NS group (NC/NS) (108 out of 1724 phosphopeptides) and ∼6.7% in HC versus NC (HC/NC) group (115 out of 1724 phosphopeptides) were significantly altered using a cut-off value of 1.5-fold up- or down-regulation (Figure 2, c and d). Among these altered phosphopeptides, 58 phosphopeptides were found in common between NC/NS and HC/HS comparison groups, in which 12 have the same alteration trend (Figure 2 e and f).

### Properties of Phosphorylated Proteins

**Figure 3:**
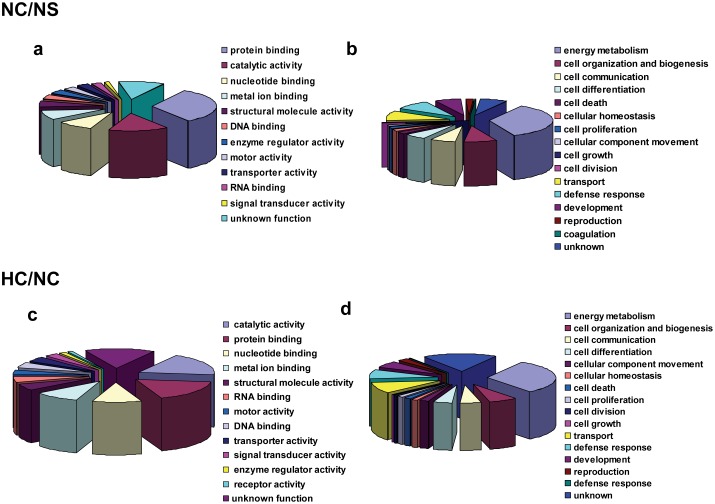
GO analysis of the phosphoproteins differentially expressed in NC/NS and HC/NC comparison groups based on their molecular function (a, c) and biological process (b, d) using PANTHER classification, respectively.

To understand biological roles of these phosphoproteins in cardiac remodeling process, a Gene Ontology (GO) analysis with PANTHER classification system was utilized to analyze molecular functions and biological process of these differentially phosphorylated proteins. As shown in Figure 3, GO analysis for NC/NS comparison group demonstrated that the differentially expressed phosphoproteins were classified into 12 groups based on their molecular functions including protein binding, catalytic activity, nucleotide binding, metal ion binding, structural molecule activity, enzyme regulator activity, DNA binding, motor activity, transporter activity, RNA binding, signal transducer activity, and 16 groups according to their biological process such as energy metabolism, transport, cell growth, cell death, cell communication, cell differentiation cell organization and biogenesis and development, (Figure 3 a and b). Similarly, GO analysis demonstrated that the differentially expressed phosphoproteins for HC/NC comparison group were classified into 13 groups based on their molecular functions including catalytic activity, protein binding, nucleotide binding, metal ion binding, structural molecule activity, RNA binding, motor activity, DNA binding, transporter activity, signal transducer activity, enzyme regulator activity, receptor activity, and 16 groups according to their biological process such as energy metabolism, transport and cell differentiation (Figure 3 c and d). Here, energy metabolism means the chemical processes occurring within a living cell or organism that are necessary for the maintenance of life. Cell organization and biogenesis means a process that results in the biosynthesis of constituent macromolecules, assembly, arrangement of constituent parts, or disassembly of a cellular component. Cell communication includes any process that mediates interactions between a cell and its surroundings. Cell differentiation means the process in which relatively unspecialized cells, e.g. embryonic or regenerative cells, acquire specialized structural and/or functional features that characterize the cells, tissues, or organs of the mature organism or some other relatively stable phase of the organism’s life history. Cell death includes any biological process that results in permanent cessation of all vital functions of a cell. Cellular homeostasis defines any process involved in the maintenance of an internal steady state at the level of the cell. Cell proliferation defines the multiplication or reproduction of cells, resulting in the expansion of a cell population. Cellular component movement means the directed, self-propelled movement of a cellular component without the involvement of an external agent such as a transporter or a pore. Cell growth defines the process in which a cell irreversibly increases in size over time by accretion and biosynthetic production of matter similar to that already present. Cell division means the process resulting in the physical partitioning and separation of a cell into daughter cells. Transport covers the processes involved in positioning a substance or cellular entity. Defense response means reactions, triggered in response to the presence of a foreign body or the occurrence of an injury, which result in restriction of damage to the organism attacked or prevention/recovery from the infection caused by the attack. Development defines the process whose specific outcome is the progression of the cell over time, from its formation to the mature structure. Reproduction means a process, occurring at the cellular level, that is involved in the reproductive function of a multicellular or single-celled organism. Coagulation means the process in which a fluid solution, or part of it, changes into a solid or semisolid mass. In addition, we have listed some known proteins that are associated with heart damage (Table 2). The involved heart diseases included hypertrophic cardiomyopathy, arrhythmogenic right ventricular cardiomyopathy, dilated cardiomyopathy and viral myocarditis.

**Table 2:** List of some identified proteins and their known associated heart disease.

Gene name	ID	Heart disease
phospholamban	IPI00195376	Dilated cardiomyopathy
desmin	IPI00421517	Hypertrophic cardiomyopathy;Arrhythmogenic right ventricularcardiomyopathy; Dilatedcardiomyopathy
myosin, heavy chain 9,non-muscle	IPI00209113	Viral myocarditis
lamin A	IPI00454367	Hypertrophic cardiomyopathy;Arrhythmogenic rightventricular cardiomyopathy;Dilated cardiomyopathy
junctophilin-2	IPI00199887	hypertrophic cardiomyopathy
catenin, alpha 1	IPI00358406	Arrhythmogenic rightventricular cardiomyopathy
desmoplakin	IPI00366081	Arrhythmogenic rightventricular cardiomyopathy
desmoglein 2	IPI00951246	Arrhythmogenic rightventricular cardiomyopathy
plakophilin 2	IPI00763527	Arrhythmogenic rightventricular cardiomyopathy
myosin bindingprotein C, cardiac	IPI00870316	Hypertrophic cardiomyopathy;Dilated cardiomyopathy
myosin, heavy chain 6,cardiac muscle, alpha	IPI00189809	Hypertrophic cardiomyopathy;Dilated cardiomyopathy;Viral myocarditis
myosin, heavy chain 7,cardiac muscle, beta	IPI00189811	Hypertrophic cardiomyopathy;Dilated cardiomyopathy;Viral myocarditis
troponin I type 3 (cardiac)	IPI00231689	Hypertrophic cardiomyopathy;Dilated cardiomyopathy

### STRING Protein-protein Analysis of Differentially Expressed Heart Proteins

**Figure 4:**
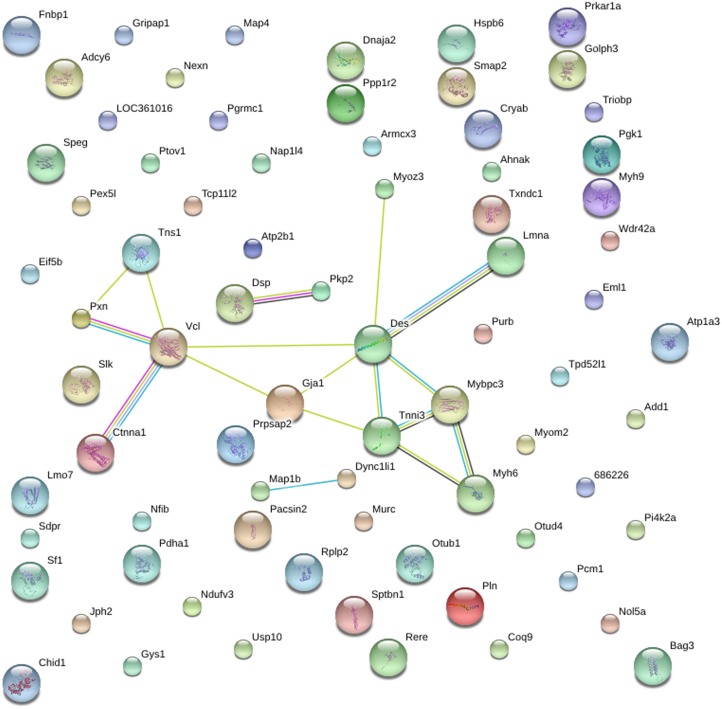
STRING analysis reveals protein interaction networks in heart phosphoproteome in NC/NS comparison group. Interactions of the identified phosphoproteins were mapped by searching the STRING (Search Tool for the Retrieval of Interacting Genes/Proteins) database version 9.0 with a confidence cutoff of 0.6. In the resulting protein association network, proteins are presented as nodes which are connected by lines whose thickness represents the confidence level (0.6–0.9).

**Figure 5:**
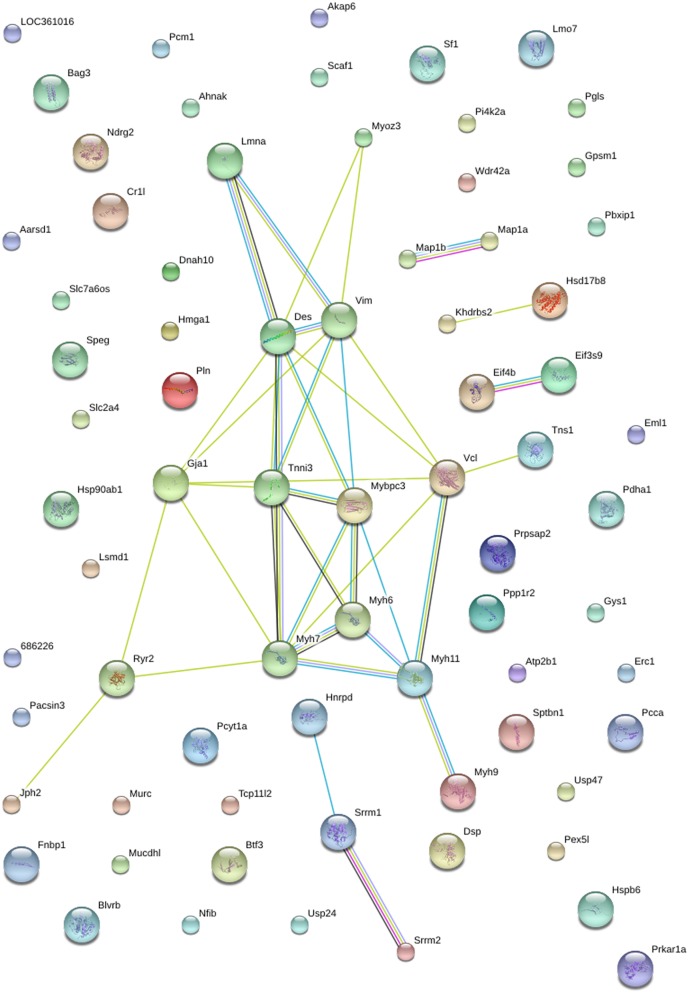
STRING analysis reveals protein interaction networks in heart phosphoproteome in HC/NC comparison group. Interactions of the identified phosphoproteins were mapped by searching the STRING database version 9.0 with a confidence cutoff of 0.6. In the resulting protein association network, proteins are presented as nodes which are connected by lines whose thickness represents the confidence level (0.6–0.9).

STRING (Search Tool for the Retrieval of Interacting Genes) is a protein-protein analysis database program generating a network of interactions from a variety of sources, including different interaction databases, text mining, genetic interactions, and shared pathway interactions [33]. This analysis provides an essential system-level understanding of cellular events in a functional heart. The networks formed by interacting proteins provided insights into the potential mechanisms of how salt and renal failure affects cardiac functions. In this study, the STRING analysis revealed functional links among 15 significantly regulated phosphoproteins in NC/NS comparison group (Figure 4) and 23 significantly regulated phosphoproteins in HC/NC comparison group (Figure 5).

### Validation of Selected Proteins and Analysis of their Downstream Gene Expression

**Figure 6:**
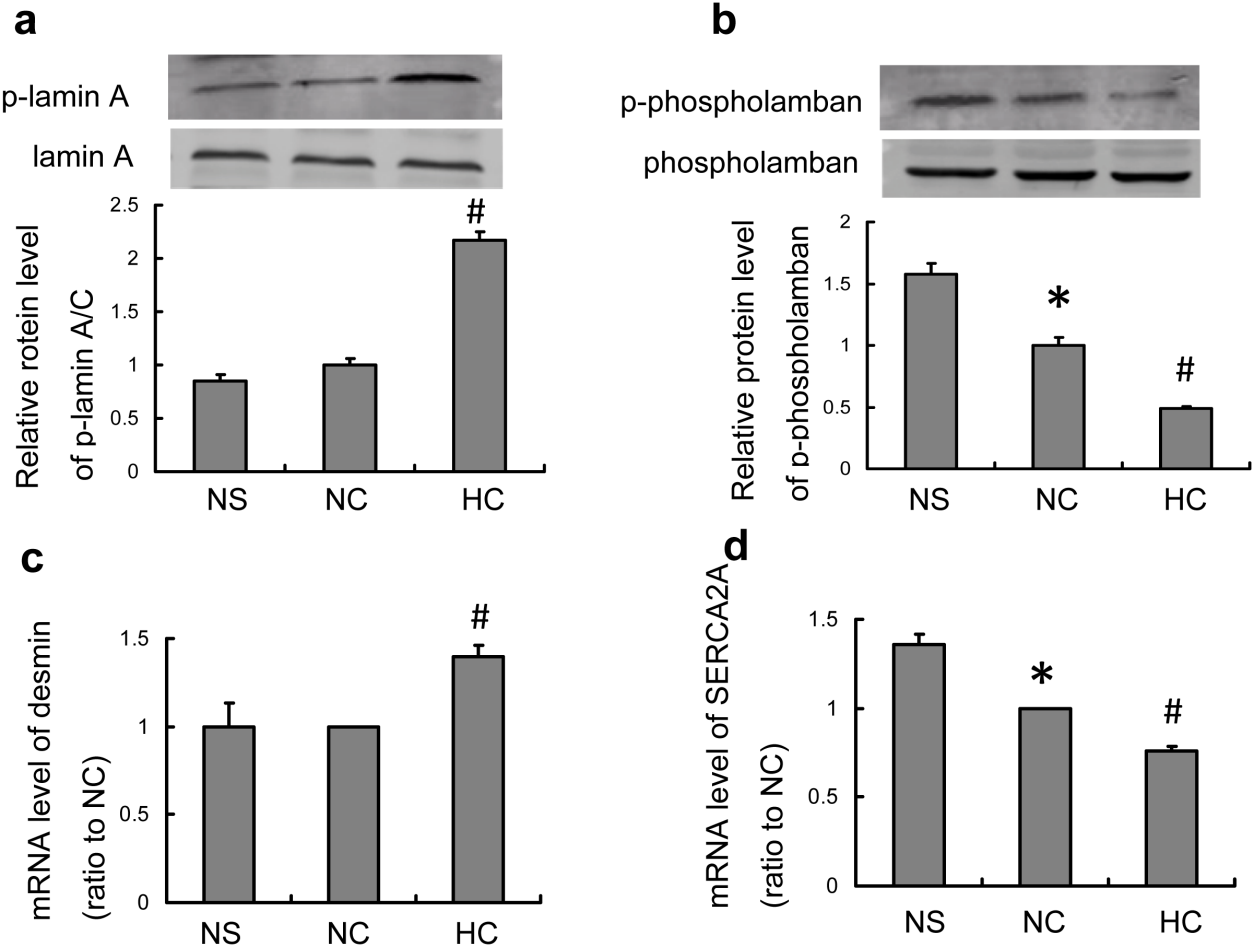
High salt intake induced significant expression changes of p-lamin A and p-phospholamban as well as their downstream genes desmin and SERCA2a. High salt intake increased protein level of p-lamin A (a) and mRNA level of its downstream gene desmin (c). Phosphorylation level of phospholamban decreased (b) and that resulted in decrease of mRNA level of the downstream gene SERCA2a (d) in NC and HC groups. *P*<0.05 vs. NS (*) and vs. NC group (#).

Given that one of the major goals of this project was to identify phosphoproteins that may be contributing to salt-induced heart damage, we examined expression of phospho-lamin A and phospho-phospholamban as well as their downstream genes desmin and SERCA2a, which altered significantly in response to high salt intake in CRF rats. We initially examined expression of phospho-lamin A by western blot. It has been shown that lamin A/C-deficient myoblasts showed a decrease in desmin protein and transcript [34]. Restoration of lamin A in cardiomyocytes improves cardiac function [35]. Consistent with our proteome analysis, we noted high salt-induced a marked increase in phospho-lamin A in CRF animals (Figure 6a). Further, a significant increase in desmin mRNA level was observed in high salt-fed CRF rats (Figure 6c), which corresponded with increased phospho-lamin A expression.

We then examined expression of phospho-phospholamban in left ventricular free walls. SERCA/phospholamban complex regulates cardiac muscle contractility by controlling Ca2+ transport in cariomyocytes. Phosphorylation of phospholamban increases SERCA expression [36]. Western analysis of left ventricular free walls revealed a significant decrease in phosphor-phospholamban expression in CRF rats (Figure 6b). High salt intake resulted in a further reduction of phosphorylated phospholamban in CRF rats (Figure 6b). Consistently, mRNA levels of its downstream gene SERCA were thus decreased in NC and HC groups (Figure 6d). Together, these data lend support to our proteomic analysis.

## Discussion

Previous studies have suggested significant changes of phosphorylated heart proteins in animal models such as spontaneously hypertensive rats [21], [37]–[39], Dahl rats [40] and heart failure model [41] or cardiac cell line [42]. Protein phosphorylation plays a critical role in regulation of cardiac function. It must be noted that we were the first to investigate the phosphorylated proteins of the heart as well as characterize the variations induced by high salt intake in the remnant kidney model.

We have identified 763 phosphorylated proteins and 1724 phosphopeptides by iTRAQ along with LC-MS/MS. Here we have demonstrated that quantitative iTRAQ-based LC MS/MS is a robust protein discovery technique, and has the potential to uncover proteins as yet unknown to function in pathogenesis of cardiovascular changes caused by CRF. Our investigation focused on identifying as many post-translational modification alterations as possible. Phosphorylation is the most prevalent post-translational modification. Titanium dioxide enrichment was performed, which has been proved highly efficient and selective for phospho-enrichment [43], as phosphosignals are frequently restrained to such an extent that they are lost to their more abundant un-modified counterparts without any enrichment methods. Therefore, post-translational modifications were specifically searched for.

We have identified many molecules associated with cardiac function. For instance, cMyBP-C, cardiac myosin-binding protein-C, is an important regulator of cardiac contractility, and its phosphorylation by PKA contributes to increased cardiac output in response to β-adrenergic stimulation [44]. cMyBP-C phosphorylation level is markedly decreased in human and animals with heart failure [45]. Similarly, we have observed cMyBP-C phosphorylation levels in high salt-fed CRF rats, suggesting an important maladaption to salt-reduced cardiac damage in CRF rats.

Phospholamban is a member of calcium signaling pathway and small transmembrane protein that is located in the cardiac sarcoplasmic reticulum. Phospholamban binds to and regulates the activity of a Ca2+ pump SERCA2a through altering its phosphorylation state. There is evidence that dilated cardiomyopathy in humans can result from chronic inhibition of SERCA2a by the prevention of phosphorylation of phospholamban by PKA [46]. In our study, proteomic data revealed that phospholamban phosphorylation level decreased significantly in CRF rat hearts, that were aggravated by salt loading. Change of phospholamban phosphorylation was validated by secondary method western blot. Importantly, a marked decrease in SERCA2a transcript was also observed here. These data may suggest dysregulation of Ca2+ pump activity and signaling. This may reveal a mechanism underlining dilated cardiomyopathy in CRF.

Junctophilin-2, a unique subtype rich in the heart, is a membrane-binding protein that plays a key role in organization of junctional membrane complexes in cardiac myocytes. It is essential for cellular Ca^2+^ homeostasis and cardiac excitation-contraction coupling. Junctophilin-2 decreased in cardiac diseases such as hypertrophic cardiomyopathy [47], [48], dilated cardiomyopathy and heart failure [47], [49], thus contributing to defective excitation-contraction coupling. In this study, phosphorylation level of junctophilin-2 was observed to decrease significantly in salt-fed CRF group, suggesting that phosphorylation of junctophilin-2 may play an important role in salt-induced cardiac injury associated with CRF.

To reveal potential signaling pathways represented by the heart phosphoproteome, we searched the identified phosphoproteins based on the widely used pathway database, Kyoto Encyclopedia of Genes and Genomes (KEGG) [50], [51]. Many fundamental biological pathways were highlighted by phosphoproteins differentially expressed in NC/NS and HC/NC comparison groups, as shown in Table S3 and S4, which included calcium signaling pathway, hypertrophic cardiomyopathy, dilated cardiomyopathy, Arrhythmogenic right ventricular cardiomyopathy, cardiac muscle contraction, MAPK signaling pathway, adherens junction, tight junction, etc. These signaling pathways may be related to differences in heart phosphoproteome of 5/6 Nx rats with different salt intake. Therefore, our phosphoproteomics data provided a deeper understanding of phosphorylation regulation and laid a foundation for future dissection of the phosphorylation network in damaged hearts due to renal failure and salt load.

## Conclusions

Our global phosphoprotein analysis based on iTRAQ identified 1724 unique phosphopeptides representing 2551 non-redundant phosphorylation sites corresponding to 763 phosphoproteins in left ventricular free walls of CRF rats. Among these phosphopeptides, 89 upregulated and 76 downregulated in CRF animals relative to sham group. Compared to normal salt intake, salt load induced upregulation of 84 phosphopeptides and downregulation of 88 phosphopeptides in CRF rats. The differentially expressed phospholproteins are important signaling molecules, receptors, phosphatases, and transcription regulators involved in energy metabolism, transport, cell organization and biogenesis, cell communication, cell differentiation, cell death and other biological processes. Although the pathological significance of differentially phosohorylated peptides remains to be tested, identification of phosphopeptide profiles involved in CRF and salt load will advance our understanding of chronic kidney disease -induced heart damage and help identify new potential therapeutic target.

## Supporting Information

Table S1 **Complete list of phosphopeptides identified from hearts in rats with chronic renal failure.**
(XLS)

Table S2 **The 279 identified peptides differentially phosphorylated in NC/NS and/or HC/NC comparison groups.**
(XLS)

Table S3 **KEGG pathways targeted by the 165 identified differentially phosphorylated peptides in NC/NS comparison group.**
(XLS)

Table S4 **KEGG pathways targeted by the 172 identified differentially phosphorylated peptides in HC/NC comparison group.**
(XLS)
